# Supplementary material for: Recalibrating timing behavior via expected covariance between temporal cues
Source: eLife. 2018 Nov 2;7:e38790. doi: 10.7554/eLife.38790 (PMC6235573; doi:10.7554/eLife.38790)
Supplement: Figure 1—source data 2. — Stars indicate significance under an alpha level of .05. Tildes indicate marginally significant effects (p < .1). [file elife-38790-fig1-data2.docx]

| Experiment | Group/Condition | Peak time | Start time | Stop time |
| --- | --- | --- | --- | --- |
| Experiment 1 | 8-to-4 | -22% +/- 5%* | -4% +/- 12% | -27% +/- 5%* |
|  | 16-to-32 | 59% +/- 9%* | 59% +/- 12%* | 65% +/- 10%* |
|  | 8-to-8 | 6% +/- 5% | 17% +/- 11% | 3% +/- 5% |
| Experiment 2 | 8-to-12 | 13% +/- 5%* | 18% +/- 10%~ | 9% +/- 3%* |
| Experiment 3 | Change | -47% +/- 4%* | -33% +/- 5%* | -46% +/- 4%* |
|  | No-change | -13% +/- 8% | -4% /- 11% | -19% +/- 5%* |
| Experiment 4a | Correlated | 13% +/- 5%* | 21% +/- 7%* | 12% +/- 4%* |
|  | Uncorrelated | -6% +/- 4% | 1% +/- 5% | -3% +/- 4% |
| Experiment 4b | Correlated | 53% +/- 22%* | 63% +/- 30%* | 47% +/- 19%* |
|  | Uncorrelated | 5% +/- 3% | 6% +/- 4% | 15% +/- 7% |
| Experiment 5 | Change context | 46% +/- 12%* | 68% +/- 21%* | 46% +/- 12%* |
|  | No-change context | 19% +/- 6%* | 38% +/- 10%* | 10% +/- 7% |

Figure 1—figure supplement 4. Table showing percent change in peak, start, and stop times (+/- SEM) for the unchanged cue across all experiments. Stars indicate significance under an alpha level of .05. Tildes indicate marginally significant effects (*p* < .1).
